# Human translingual neurostimulation alters resting brain activity in high-density EEG

**DOI:** 10.1186/s12984-019-0538-4

**Published:** 2019-05-27

**Authors:** Zack Frehlick, Bimal Lakhani, Shaun D. Fickling, Ashley C. Livingstone, Yuri Danilov, Jonathan M. Sackier, Ryan C. N. D’Arcy

**Affiliations:** 1 HealthTech Connex, Surrey, British Columbia Canada; 2 Pavlov Institute of Physiology, Russian Academy of Science, Sankt Petersburg, Russia; 3 Helius Medical Technologies, Newtown, PA USA; 4 Oxford University, Oxford, UK

**Keywords:** Cranial nerve stimulation, Neuromodulation, Neuroplasticity, EEG

## Abstract

**Background:**

Despite growing evidence of a critical link between neuromodulation technologies and neuroplastic recovery, the underlying mechanisms of these technologies remain elusive.

**Objective:**

To investigate physiological evidence of central nervous system (CNS) changes in humans during translingual neurostimulation (TLNS).

**Methods:**

We used high-density electroencephalography (EEG) to measure changes in resting brain activity before, during, and after high frequency (HF) and low frequency (LF) TLNS.

**Results:**

Wavelet power analysis around Cz and microstate analysis revealed significant changes after 20 min of stimulation compared to baseline. A secondary effect of exposure order was also identified, indicating a differential neuromodulatory influence of HF TLNS relative to LF TLNS on alpha and theta signal power.

**Conclusions:**

These results further our understanding of the effects of TLNS on underlying resting brain activity, which in the long-term may contribute to the critical link between clinical effect and changes in brain activity.

## Background

A growing body of evidence suggests that translingual neurostimulation (TLNS) (i.e., neuromodulation) may facilitate neuroplasticity-related changes in the brain [1, 2]. When coupled with targeted therapy in clinical trials, neuromodulation has resulted in significant improvements on clinical measures. Due to the potential clinical impact of neuromodulation for neurorehabilitation of conditions such as stroke [1], mild traumatic brain injury (mTBI) [2], and multiple sclerosis [3], further studies into the physiological mechanisms involved in neuromodulation therapy are of significant interest.

One such neuromodulation device is the Portable Neuromodulation Stimulator (PoNS®; Helius Medical Technologies: Newtown, PA, USA), an investigational medical device, which involves sequenced noninvasive stimulation applied to the tongue. It is generally hypothesized in the existing literature that the tongue stimulation engages the trigeminal (CN-V) and facial (CN-VII) cranial nerves, which converge on and co-modulate visual, vestibular, nociceptive and visceral sensory signals via the brainstem and cerebellum, producing a neuromodulatory effect [3–5]. Recent evidence suggests that the trigeminal nerve is involved in networks of activity which affect sensorimotor and cognitive functions, and that modulation may relieve symptoms of particular brain pathologies [6].

In clinical trials, PoNS® therapy has included both high frequency (HF) and low frequency (LF) stimulation levels. These stimulation modalities are identical in terms of form and usage. LF stimulation involves a significantly lower frequency of stimulation and has therefore been used as a relative experimental control comparison measure. However, recent unpublished clinical trial results suggest that both HF and LF stimulation produced positive recovery outcomes for individuals with mTBI. It appears that both stimulation levels may influence brain function recovery, but the difference in effect and the underlying neurophysiological mechanisms are not well understood. A critical link is needed between improved functional recovery and changes in brain activity.

Accordingly, the current study used high-density electroencephalography (EEG) to measure brain activity before, during, and after PoNS® use [7]. We investigated whether a single 20-min session of PoNS® would elicit significant EEG changes in brain activity using a pre- post- comparison (i.e., after PoNS® was completed and could not directly contribute to EEG signal changes). The study hypothesis predicted that significant changes would occur in two EEG measures: spectral frequencies and spatial microstates. In addition, we investigated whether HF and LF stimulation levels produce different patterns of EEG spectral frequencies and/or spatial microstates. Spectral analysis was chosen because it is a common technique for identifying generalized activity changes. Microstate analysis was chosen because it is an emerging technique for quantifying large-scale spatial network patterns in resting state EEG.

## Methods

**Fig. 1:**
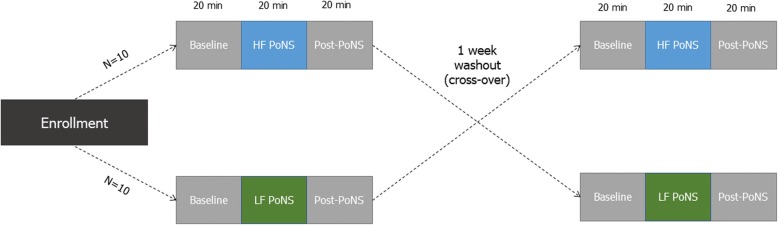
Design of the counterbalanced cross-over study

Twenty participants (*N* = 20) were enrolled and consented in an IRB approved and Health Canada approved research protocol. All participants received both the HF and LF PoNS® in a counterbalanced cross-over within-subjects design (Fig. 1). The PoNS® device delivers stimulation to the anterior dorsal tongue via 143 gold-plated electrodes. Electrodes with 1.5 mm diameter are positioned in a hexagonal pattern with 2.2 mm center-center spacing [8]. The electrode array is held in contact with the tongue by pressure from the jaw and mouth of the wearer.

PoNS® stimulation levels are represented as discrete values from 1 to 60, indicating the length of stimulation pulses (in μs). The HF device delivered triplets of pulses at 5 ms intervals (i.e., 200 Hz) every 20 ms (50 Hz), while the LF device delivered a single pulse every 781 ms (1.28 Hz). The rated operating voltage and current of each pulse are 17.5 V and 440 μA, respectively. Due to individual variations in tolerance for the electrical stimulus, stimulation levels were individually determined during the study according to a level setting procedure provided by the device manufacturer. At least 24 h prior to each EEG recording session, participants briefly tested the device (either HF or LF) by using control buttons to increase stimulation to the threshold of sensation (minimum perceptible level) and the threshold of discomfort (maximum tolerable level). Individual stimulation levels were then fixed at 75% of the difference between minimum and maximum. This procedure, established based on empirical data from psychophysical studies, ensures that the tactile sensation is strong and readily sensed, yet comfortable for long-term use [8, 9]. See Table 1 for a summary of stimulation levels received by individual subjects, in terms of stimulation level and charge delivered (μC/s).

**Table 1:** Summary of stimulation levels for individual participants

Subject ID	Stimulation on 1st Day of Crossover Trial	HF PoNS Stim. Level	HF PoNS Charge Delivered (μC/s)	LF PoNS Stim. Level	LF PoNS Charge Delivered (μC/s)	Excluded from Signal Power Analysis	Excluded from Microstate Analysis
1	HF	24	226.5	47	0.2366		
2	LF	15	141.6	47	0.2366		
3	HF	20	188.8	47	0.2366		
4	LF	11	103.8	46	0.2315		
5	HF	46	434.1	48	0.2416		
6	HF	46	434.1	48	0.2416		
7	LF	21	198.2	47	0.2366		
8	HF	22	207.6	49	0.2466		
9	LF	10	94.4	46	0.2315		
10	HF	17	160.4	47	0.2366	X	X
11	HF	17	160.4	47	0.2366		
12	LF	9	84.9	42	0.2114	X	X
13	HF	11	103.8	48	0.2416		
14	LF	26	245.4	46	0.2315		X
15	HF	13	122.7	48	0.2416		
16	LF	19	179.3	46	0.2315		
17	HF	27	254.8	41	0.2064		
18	LF	5	47.2	30	0.1510		
19	HF	17	160.4	47	0.2366		
20	LF	8	75.5	43	0.2164		

Neural activity was measured before, during, and after stimulation using a 64-channel high-density ActiCAP EEG system (Brain Products: Munich, Germany). EEG activity was recorded using Ag-AgCl electrodes, with impedance at or below 20 kΩ, sampling at 1000 Hz, and a 500 Hz lowpass filter. During each session, participants underwent three periods of testing: (1) baseline (20 min of EEG recording at rest); (2) stimulation (20 min of EEG recording with the PoNS®); and (3) post-stimulation (20 min of EEG recording at rest). During each 20-min session, the participants completed breathing and awareness training, as directed by an audio recording in order to maintain attention.

Raw EEG data were pre-processed using Brain Vision Analyzer (Brain Products: Munich, Germany) by applying a common average reference, bandpass filtering between 0.1 and 100 Hz (60 Hz notch), and resampling to 250 Hz. Recordings were manually analyzed and periods of data with poor signal quality or high occurrence of artifacts were rejected. On average, 243 s of data were rejected for each 20-min EEG recording. Due to individual variations in artifact frequency during EEG recording, the average amount of data rejected for each participant varied from 70 to 400 s per 20-min recording (median 247 s). Equivalent periods of data were rejected from recordings for HF stimulation days and LF stimulation days. Independent component analysis was applied using EEGLAB [10] to further remove persistent ocular and electrical artifacts. Data from two participants had consistently poor signal quality and were not included in subsequent analyses. One rejected participant received LF stimulation on the first day of the cross-over trial and the other received HF stimulation first.

Processed data were examined to identify differences between HF and LF stimulation. Two principal analyses were conducted: time-frequency (wavelet) power analysis and spatial microstate analysis. Time-variant wavelet power was calculated for a group of electrodes around Cz to characterize activity changes within specific EEG bands. EEG time series for five electrodes (Cz, FC1, FC2, C1, C2) were averaged together prior to wavelet analysis. This centralized group of electrodes was chosen because spectral changes were hypothesized to be general and widespread. Other electrode groups were initially investigated and found to be qualitatively similar, so further analysis focused on the central group. To quantify the wavelet analysis, data were split into four common frequency bands: alpha (8–12 Hz), theta (4–8 Hz), beta (12–30 Hz), delta (2–4 Hz). Further analysis focused in alpha and theta bands as these are most related to the resting-state breathing and awareness training task.

Spatial microstate analysis was conducted according to published methods [11, 12] to assess functional activity changes in the resting state EEG. Microstate analysis involves characterizing EEG as a series of discrete quasi-stable activity patterns. For each EEG recording, pre-processed data were filtered from 2 to 20 Hz and the global field power (GFP) time series was computed (the standard deviation of all electrode values at each time sample). Local GFP maxima were marked as microstate timepoints, and the 64-channel data were sampled at these timepoints. All 64-channel samples from each recording session (baseline, during stimulation, post-stimulation) were passed through a modified k-means clustering algorithm [13] and tagged as one of four discrete states (referred to as A, B, C, D). Secondary statistics for these discrete states were then computed and plotted: frequency of occurrence, average duration of each occurrence, and coverage (proportion of time spent in each state). Data from one additional participant could not be successfully decomposed and was subsequently omitted from the analysis. This participant received LF stimulation on the first day of the cross-over trial.

Main effects of time (baseline/post-PoNS®) and stimulation type (HF/LF) were assessed using repeated measures analyses of variance (ANOVA) with respect to signal power and spatial microstates. Unplanned post-hoc sub-analysis on exposure order effects of HF vs. LF stimulation was conducted using a subsequent repeated measures ANOVA with main effects of group (HF First/LF First), stimulation type (HF/LF) and time (baseline/post-PoNS®) for both the signal power and spatial microstates. For signal power sub-analysis, group sizes were 10 HF First and 8 LF First. For microstate sub-analysis, group sizes were 10 HF First and 7 LF First. In the event of a significant interaction effect, pairwise comparisons were conducted using Tukey tests. For the microstate analysis, in addition to testing the absolute changes in microstate metrics (duration and coverage) using ANOVA, paired t-tests were used to compare normalized data (% change relative to baseline) with the baseline mean within each testing session. All statistical comparisons were made based on average values for 20-min recordings (i.e. average alpha power at baseline was compared with average alpha power post-stimulation). Independent samples t-tests were also conducted to compare stimulation levels between groups (HF First/LF First) for both HF and LF stimulation. Statistical analysis was conducted using SPSS Statistics, Subscription Software (IBM; Armonk, NY, USA). The critical alpha for all statistical tests was *p* ≤ 0.05.

## Results

### Signal power analysis

**Fig. 2:**
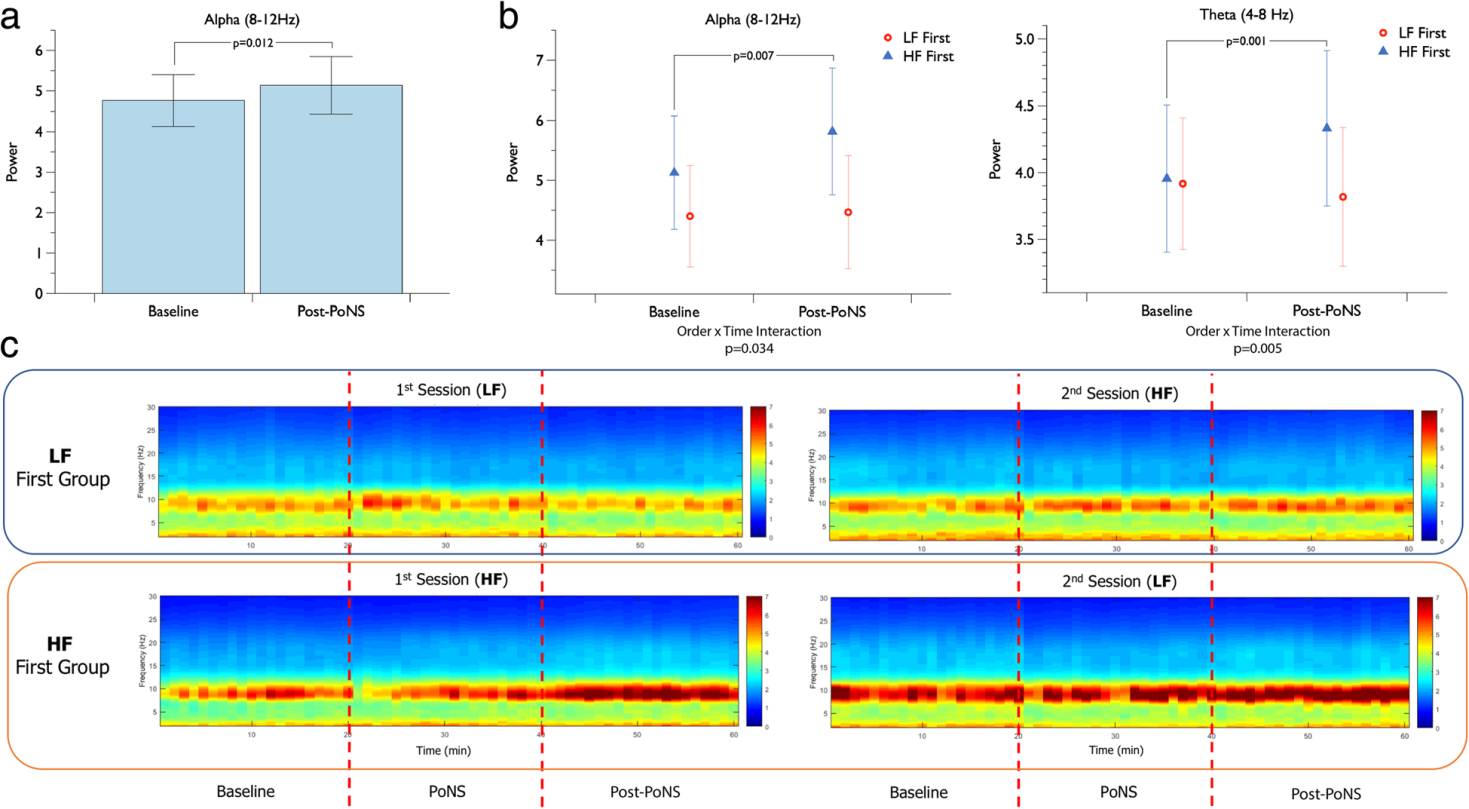
**a** Comparison of alpha EEG power before and after PoNS®, displaying statistically significant main effect of time; (**b**) Order by time interaction effect on alpha and theta power and Tukey pairwise post-hoc test statistical tests demonstrating a statistically significant increase alpha and theta power when exposed to HF stimulation in the first session; (**c**) Time-frequency spectral power for each exposure group (HF First and LF First) during each testing session

Wavelet power analysis of electrodes around Cz demonstrated a statistically significant main effect of time (F_1,16_ = 7.965, *p* = 0.012) on alpha brain activity (Fig. 2a), indicating that alpha (8–12 Hz) EEG patterns were more prominent following 20 min of PoNS® usage. The lack of interaction or main effect of stimulation type demonstrate that the result collapsed across both HF and LF stimulation conditions, and therefore represented an average increase in alpha signal power across experimental conditions.

Subsequently, a sub-analysis on order exposure effects revealed a significant interaction effect between group (HF First/LF First) and time in the alpha and theta power spectra (F_1,16_ = 5.402, *p* = 0.034 and F_1,16_ = 10.358, *p* = 0.005, respectively; Fig. 2b). Post-hoc testing showed that individuals exposed to HF PoNS® in their first session demonstrated significantly increased alpha (*p* = 0.007) and theta (*p* = 0.001) brain activity within both HF and LF PoNS® sessions, relative to the baseline of that session (Figs. 2b and c). Individuals exposed to LF PoNS® in their first session did not show significant activity changes after PoNS® usage in either session.

### Microstate analysis

**Fig. 3:**
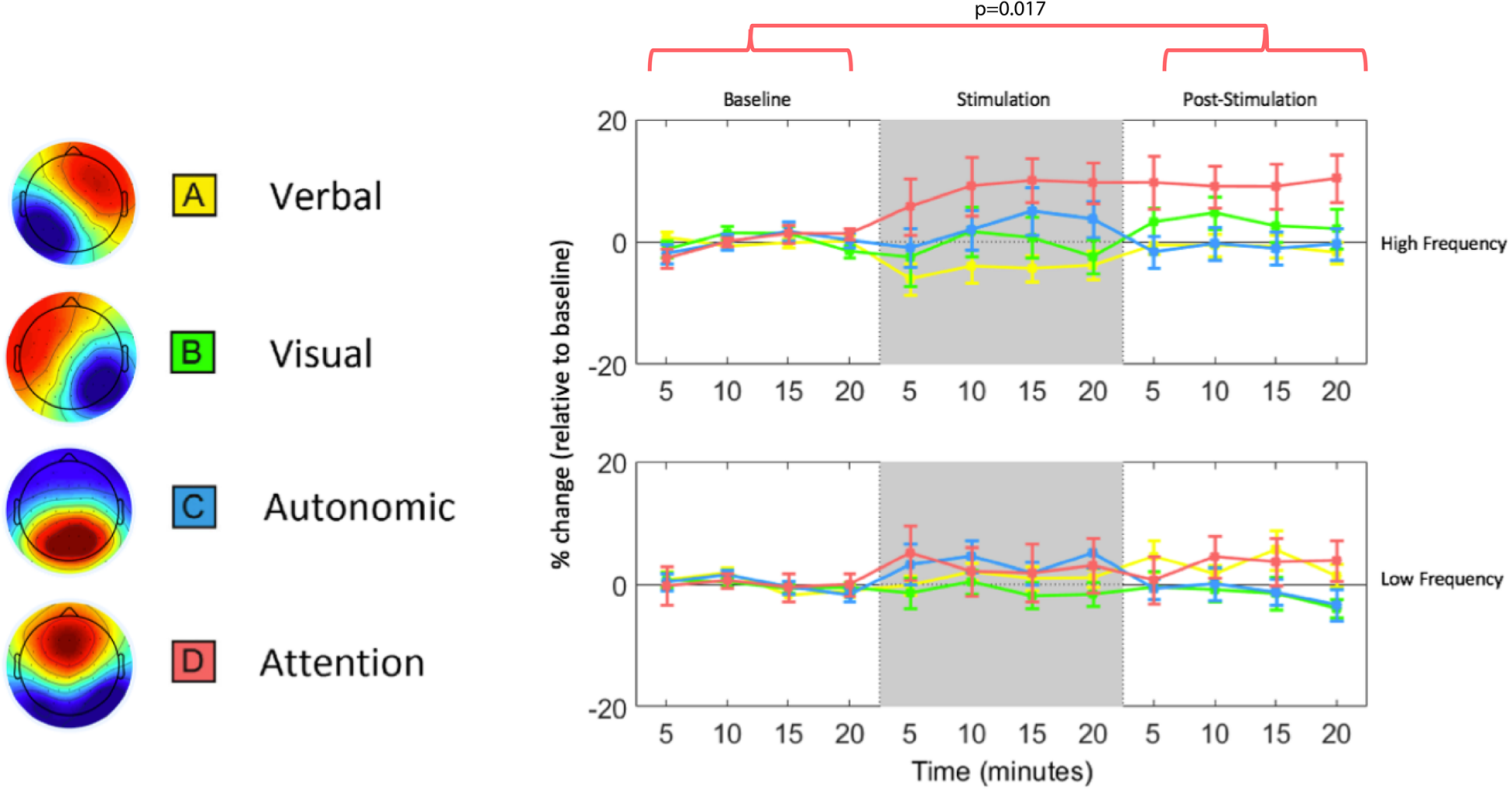
(Left) Spatial topography of EEG microstate activation (adapted from ^9^). (Right) Average (± standard error) microstate duration during each testing phase (binned into 5-min intervals). There was a statistically significant increase in the normalized duration of microstate D (attention) compared to baseline

There were no statistically significant main effects of session or stimulation type on any of the four microstates when tested using ANOVA (Fig. 3). The sub-analysis on order exposure effects revealed no significant effect of HF/LF order exposure on any of the microstate durations. Normalized microstate analysis tested by paired t-test revealed a statistically significant increase in duration of microstate D (associated with attention) following HF stimulation when compared to baseline (t_16_ = 2.677, *p* = 0.017). There were no significant microstate changes found following LF PoNS® usage. No statistically significant interaction effects were found between order of HF/LF exposure and changes in microstate activity.

### Stimulation level analysis

For HF stimulation, independent samples t-tests demonstrated a statistically significant difference in stimulation level between groups (HF First/LF First; t_18_ = 2.182, *p* = 0.043). In contrast, for LF stimulation, there was no statistically significant difference in stimulation level between groups.

## Discussion

To our knowledge, the current results represent the first demonstration that neuromodulation significantly changes brain activity. The findings may provide insight into results from previous multi-site clinical trials, which have demonstrated clinical improvements after exposure to both the HF and LF PoNS® devices.

Wavelet power analysis revealed a relationship between the HF/LF intensity and the order of exposure. Specifically, participants showed no significant increase in alpha and theta signal power if they received LF PoNS® stimulation first. Whereas, there was a significant increase in alpha and theta signal power if they received HF PoNS® stimulation first. It is noteworthy that the latter result demonstrated an increase for LF PoNS® stimulation, only if HF PoNS® stimulation occurred first. Order of exposure also affected individuals subjective intensity tolerances, with those that received HF PoNS® first tolerating a significantly higher self-selection for the stimulation level. Overall, the results suggest that the relationship between the brain and the two PoNS® stimulation levels is complex, and more research is needed to characterize PoNS® intensity and exposure effects on EEG-based neural activity.

While the wavelet analysis focused on a group of electrodes around Cz, a spatial microstate analysis was used to investigate spatial pattern changes. Microstate analysis characterizes EEG activity in terms of dynamically changing ‘building blocks’ associated with ongoing mental processes [14, 15]. HF stimulation particularly appears to promote microstate D, associated with attentional sub-systems in the brain. Increased time spent in an attentional EEG microstate has been associated with focus switching and relaxed wakefulness [12]. Conversely, decreases in the attentional microstate duration have been observed in schizophrenia [16] and during sleep [17]. Microstate effects suggest that even during rest, the PoNS® may elicit functional changes in the brain that are associated with evidence of increased neuroplasticity-related improvements.

The current study has important limitations. The single-session crossover design means that the effects of repeated stimulation cannot be evaluated. Each participant used a single individualized HF stimulation level and a single individualized LF stimulation level during the study, with variance between individuals. Future research should investigate the differences between self-selected and prescribed stimulation levels to better understand the neuro-mechanistic effect of PoNS® stimulation. Also, TLNS gives a perceivable sensation to the participant which would be difficult to imitate as a sham condition. Any perceivable level of TLNS may have a non-neutral effect on the brain, meaning that a true sham condition may not be feasible.

Finally, the study involved healthy participants engaging in passive breathing and awareness training, which does not reflect the intended use of the PoNS® for individuals with neurological conditions during task-specific therapy. This study therefore did not assess the neural effects of the PoNS® during therapeutic use. Nevertheless, these results do further our understanding of the effects of PoNS® usage on underlying resting brain activity, which in the long-term may contribute to the critical link between clinical effect and changes in brain activity. Further research could investigate (short-term) PoNS®-related changes in brain activity in individuals with mTBI.

## Conclusion

Neuromodulation, such as through the PoNS®, has been linked to improved functional outcome in brain injury and disease, but the underlying neural mechanisms remain elusive. We report the initial findings of EEG changes in resting brain activity after a single 20-min session of PoNS®. While both HF and LF PoNS® dosage levels produced significant changes in alpha and theta wave activity, HF stimulation showed differential dosage effects. HF PoNS® also significantly increased attentional microstates suggesting a possible functional mechanism associated with evidence of neuroplasticity improvements. Overall, these findings support continued characterization of the underlying neural mechanisms related to the use of neuromodulation to drive recovery of function through neuroplasticity.

## Abbreviations

ANOVA: Analysis of Variance
CN: Cranial Nerve
CNS: Central Nervous System
EEG: Electroencephalography
GFP: Global Field Power
HF: High Frequency
LF: Low Frequency
mTBI: Mild Traumatic Brain Injury
PoNS®: Portable Neuromodulation Stimulator
TLNS: Translingual Neurostimulation
